# When Emerging Fungal Infections Mimic Tuberculosis: First Reported Case of Invasive *Emergomyces europaeus* Infection in a Man With Immunocompromise Living in France

**DOI:** 10.1093/ofid/ofaf621

**Published:** 2025-10-06

**Authors:** Claire Cottrel, Natesan Ramsamy, Emilie Fruquière, Agnes Melone, Fanny Lanternier, Dea Garcia-Hermoso, Anne Debourgogne

**Affiliations:** CHRU-Nancy, Microbiologie, Université de Lorraine, Nancy, France; SIMPA, Université de Lorraine, Nancy, France; CHRU-Nancy, Maladies Infectieuses, Université de Lorraine, Nancy, France; Mycology Department, Institut Pasteur, Paris Cité University, National Reference Center for Invasive Mycoses and Antifungals, Translational Mycology Research Group, Paris, France; CHRU-Nancy, Biopathologie, Université de Lorraine, Nancy, France; Mycology Department, Institut Pasteur, Paris Cité University, National Reference Center for Invasive Mycoses and Antifungals, Translational Mycology Research Group, Paris, France; Mycology Department, Institut Pasteur, Paris Cité University, National Reference Center for Invasive Mycoses and Antifungals, Translational Mycology Research Group, Paris, France; CHRU-Nancy, Microbiologie, Université de Lorraine, Nancy, France; SIMPA, Université de Lorraine, Nancy, France

**Keywords:** *Emergomyces europaeus*, emergomycosis, molecular diagnosis

## Abstract

Emergomycosis is an emerging thermally dimorphic fungal infection primarily observed in individuals with HIV and is associated with cutaneous, pulmonary, or disseminated manifestations. We report the first case of invasive infection by *Emergomyces europaeus* involving both the lungs and bone marrow in a person with immunocompromise, identified through molecular tools.

## Teaser

We report the first documented case of invasive infection by *Emergomyces europaeus* involving both pulmonary and bone marrow sites in a man with immunocompromise, diagnosed through molecular techniques. This case is remarkable for its epidemiological rarity, its clinical presentation mimicking tuberculosis, the diagnostic complexity requiring advanced molecular tools, and the innovative therapeutic approach using novel antifungal agents.

The genus *Emergomyces,* recently reclassified using molecular tools, comprises diverse thermally dimorphic onygenalean fungi formerly classified as *Emmonsia* species. Members of this genus are characterized by small oval yeast cells with narrow-based buds, large cells with no budding (adiaspores), and adiaspore-like cells, which exhibit broad-based budding. To date, seven species have been described within *Emergomyces* [1]: *E. africanus, E. canadensis, E. pasteurianus, E. orientalis,* and *E. europaeus,* as well as species formerly associated with adiaspiromycosis, namely, *E. crescens* and *E. soli*. Emergomycosis is a rare opportunistic infection, with fewer than 80 proven cases reported [2] and an estimated burden of a hundred cases per years in South Africa [3]. A majority of documented emergomycosis cases involve *E*. *africanus* infection in people living with advanced HIV in South Africa, an endemic region [3]. Limited murine data exist with *E. africanus* disseminated infections [4]. Only a restricted number of isolated cases caused by other *Emergomyces* species have been reported outside Africa, including in North America [5], India [6], China [6] and France [7]. To date, only one case of *E. europaeus* infection has been reported in a German patient [8]. *Emergomyces* species exist as molds in the environment and are found primarily in soil and plant debris. The mycelial hyphae of these fungi produce conidia, which become aerosolized upon soil disruption. When inhaled by humans, a temperature shift induces a morphological transition to the pathogenic yeast form. The yeast cells enter the bloodstream, leading to disseminated disease, particularly in person with immunocompromise [9].

We present the first case of *E. europaeus* infection in an individual with immunocompromise in France.

## CASE

**Figure 1.**
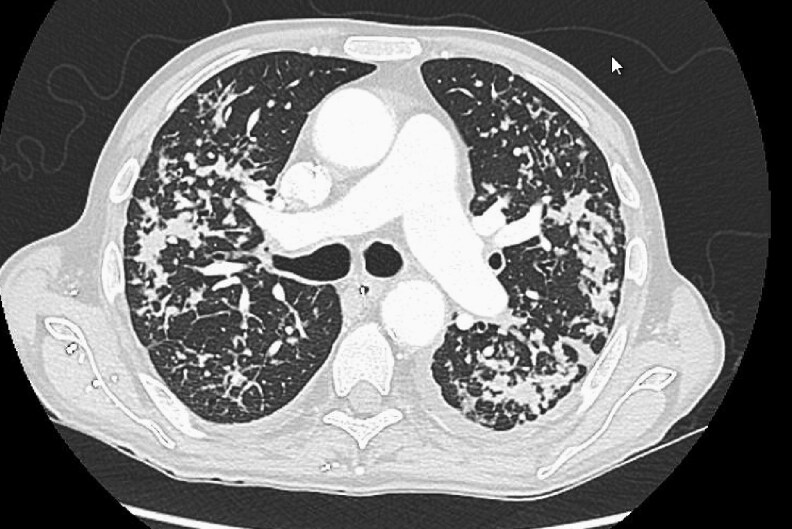
CT scan with a significant pulmonary miliary pattern.

A 65-year-old man with a three-month history of overall decline, characterized by a 10 kg weight loss, cough, and dyspnea but no fever, was admitted to the infectious disease department at a tertiary university hospital addressed by his kidney specialist. Born in Armenia, he has lived in France for 15 years and regularly travels back to Armenia. His medical history included treated pulmonary tuberculosis, cirrhosis secondary to hepatitis C, and a kidney transplant secondary to cryoglobulinemia. His immunosuppressive treatment consisted of a monthly dose of belatacept, mycophenolate mofetil, and 5 mg of prednisone daily. A Computed Tomography scan performed shortly after admission revealed a significant pulmonary miliary pattern (Figure 1). Tuberculosis was initially suspected, and samples were collected for mycobacterial diagnosis (including PCR, direct examination and culture) on a bronchoalveolar lavage (BAL) and blood culture using Myco/F Lytic culture vials.

One week later, the patient developed pancytopenia (with a leukocyte count of 1.6 G/L, a platelet count of 44 G/L and a hemoglobin level of 9.4 g/dL). The samples remained negative for tuberculosis and no positive blood culture was detected. BAL was performed, and the samples were sent to the mycology laboratory. Direct examination did not reveal any fungal elements. After two weeks of incubation, a strain grew on chromogenic medium with an atypical granular texture of the colony in macroscopy and septate hyphae with spores in microscopy but mass spectrometry tools failed to identify the fungus. Malt extract agar (MEA) medium remained sterile at day 21. With respect to biomarkers, galactomannan tests on BAL fluid and serum samples were negative (respectively 0.19 and 0.04 with Platelia BioRad® test), as were tests for serum ß-1-3 D glucans (6 pg/mL with Fujifilm Wako® test). However, the culture supernatants of the isolated strains were galactomannan positive.

**Figure 2.**
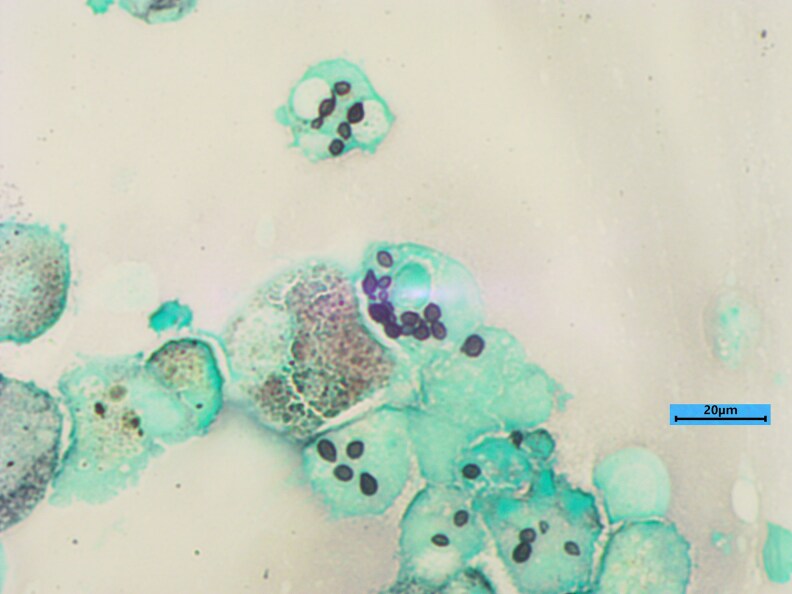
Direct examination of BAL fluid with Gomori-Grocott staining revealed small intracellular narrow-based budding yeasts.

Moreover, given the unidentified etiology and persistence of symptoms, a second BAL was performed since a pulmonary biopsy was not feasible. The immunosuppressive treatment was discontinued, and the patient required oxygen support. In the mycology laboratory, direct examination of the second BAL revealed numerous small intracellular narrow-based budding yeasts measuring 3 to 5 µm in length, as observed via May Grunwald Giemsa and Gomori‒Grocott staining (Figure 2). PCR and Sanger sequencing of the ITS1‒ITS2 region were performed in parallel directly on the second BAL sample and on the unidentified strain in culture derived from the first BAL. The result was the identification of either *Emergomyces* or *Emmonsia* species leading to a diagnosis of emergomycosis. A bone marrow biopsy was performed to investigate pancytopenia revealed macrophagic activation syndrome. After one month of incubation, the same atypical fungus was also identified in the bone marrow culture.

**Figure 3.**
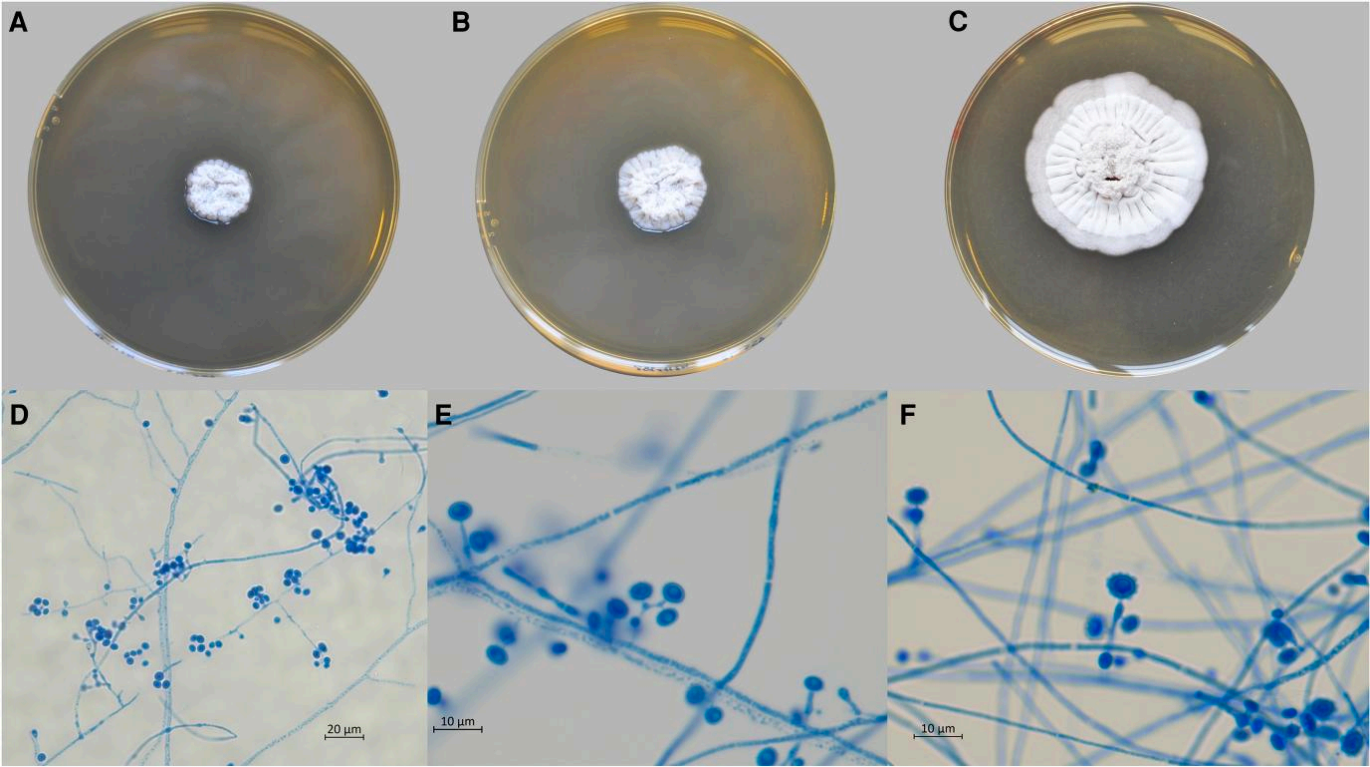
Morphological aspects of *E. europaeus* grown on 2% MEA. MEA at 30°C for 15 (A), 20 (B) or 30 (C) days. Microscopic analysis of 3-week-old microcultures (D,E,F) (30°C, MEA) revealed unbranched conidiophores distinctly swollen at the top and bearing one or two subspherical conidia.

The strain was then sent to the National Reference Center for Invasive Mycoses & Antifungals for characterization and antifungal susceptibility testing. On MEA, at 25°C and 30°C, the colony showed compact mycelia in an irregular form. After three weeks, slide cultures on MEA and Sabouraud agar at 30°C revealed enlarged conidiophores with small, roughened conidia (Figure 3). The sequencing of the ITS1-5.8S-ITS2 region of the ribosomal DNA confirmed the identification of *Emergomyces europaeus.* The percent identity with the type culture sequence UAMH 10427 was 99.83% (603/604 bp). The ITS1-5.8S-ITS2 sequence was submitted to the NCBI database with the accession number GenBank PV34149.

Antifungal susceptibility testing of the mould phase was performed following EUCAST methodology [EUCAST method for susceptibility testing of moulds (version 9.4 valid from 1 April, 2022)], and the minimal inhibitory concentrations (MICs; in mg/L) were as follows: amphotericin B, 0.125; itraconazole, 0.25; voriconazole, 0.06; posaconazole, 0.06; isavuconazole, 0.5; caspofungin, 0.007; micafungin, 0.007; and terbinafine, 0.03.

Combined therapy with liposomal amphotericin B (3 mg/kg/d) and isavuconazole (200 mg/d) was initiated following diagnosis. One week later, the pancytopenia slowly decreased, and the patient experienced clinical improvement. After 23 days of combined therapy, liposomal amphotericin B was discontinued. A follow-up Positron Emission Tomography scan 50 days after admission revealed stability of the pulmonary lesions. A consolidated treatment with posaconazole for one year was prescribed (300 mg/d), along with the resumption of immunosuppressive therapy.

## CONCLUSIONS

We present the first case of invasive *E. europaeus* infection in an person with immunocompromise. Cases of emergomycosis are extremely rare in France. Only two cases of pulmonary emergomycosis have been reported: one due to *E. pasteurianus* in an person living with HIV [7] and the other involving *E. crescens* in a patient with underlying lymphopenia in the same region (Lorraine) in eastern France [10]. Regarding *E. europaeus* in particular, a single case has been described in a patient without any immunosuppressive factors in the Swabian Alb region of southwestern Germany [8]. Geographically, these two cases are relatively close, occurring <300 km apart on either side of the Rhine River. This observation might suggest a specific focus or reservoir in this area of Europe. However, the infection may have been acquired in Armenia since the incubation period is poorly understood.

Our patient had immunosuppressive factors, including a history of solid organ (kidney) transplantation and the use of immunosuppressive drugs. In a series of 77 cases of emergomycosis, these risk factors were found in 7.8% and 13.0% of the cases, respectively, whereas HIV infection constituted the predominant risk factor (79.2%) [2]. Recent clinical studies also reported pulmonary emergomycosis in kidney transplant patients, with other species involved, such as *E. pasteurianus* and *E. orientalis* [11, 12]. The clinical presentation mimicked that of histoplasmosis or tuberculosis: the patient presented with pulmonary miliary involvement and bone marrow spread. The clinical presentation was, therefore, disseminated, whereas the other case described with *E. europaeus* infection showed isolated pulmonary localization [8]. No dermatologic symptoms were reported in our patient. Cutaneous manifestations are more frequently observed in people living with HIV, whereas pulmonary manifestations are more common in non-HIV-infected people [2]. Blood dissemination is more frequent in person living with HIV, and bone marrow dissemination is observed in this population, accounting for 18.2% of cases [2], or in the context of systemic lupus erythematosus [13].

The mycological diagnosis of this invasive infection is challenging given that three-quarters of the patients with emergomycosis are misdiagnosed with tuberculosis [14]. A combination of histopathology, fungal culture, and molecular techniques are required for accurate identification and diagnosis. However, metagenomic next-generation sequencing techniques appear promising and have recently enabled diagnosis, overcoming the limitations of conventional diagnostic methods [12, 15].

Regarding management, the treatment recommendations proposed by the ECMM in 2021 [16] were based on approaches used for histoplasmosis with amphotericin B followed by itraconazole. No reported cases have been treated with isavuconazole alone or in combination with other agents [2].
